# Nutritional Interventions in Older, Frail Persons with Heart Failure—A Systematic Narrative Review

**DOI:** 10.14283/jarlife.2024.15

**Published:** 2024-11-05

**Authors:** K. Belqaid, G.F. Irving, N. Waldréus

**Affiliations:** 1. Department of Medicine Huddinge, Karolinska Institutet, Stockholm, Sweden, and Medical Unit Health Professionals, Karolinska University Hospital, Stockholm, Sweden; 2. Department of Neurobiology, Care sciences and Society, Division of Clinical Geriatrics, Karolinska Institutet, Stockholm Sweden; 3. Department of Neurobiology, Care sciences and Society, Division of Nursing, Karolinska Institutet, Stockholm Sweden

**Keywords:** Heart failure, frailty, nutrition therapy, malnutrition

## Abstract

Frailty is a clinical condition common among older persons with heart failure (HF) and has been associated with an increased risk of adverse outcomes such as falls, disability, long-term care, and death. Malnutrition in terms of weight loss and sarcopenia is closely related to frailty. This review summarises nutritional interventions to improve components of frailty in older persons with HF. The online databases of Medline, Embase, Web of Science and Cinahl were searched in 2022 to identify studies of nutritional interventions among older persons with HF with outcomes related to frailty (e.g., body composition or functional measures). The records were screened, and eligible articles identified. In addition, reference lists of eligible articles and of four previously published reviews regarding HF and nutrition were screened. Eight articles were included in the review, of which seven were controlled trials and one was a feasibility study. Nutritional interventions included: vitamin D supplementation (n =2), protein supplementation (n =3), enteral nutrition (EN) or oral nutritional supplements (ONS) (n =2), or a low carbohydrate diet (n =1). The studies using protein supplementation, ONS or EN reported improvements on functional measures or body composition. Furthermore, the results from this review add to the evidence of the importance of combining nutritional support with physical activity to improve muscle mass and functional outcomes among older persons with HF.

## Introduction

**H**eart failure (HF) is a clinical syndrome that implies elevated intracardiac pressures and/ or inadequate cardiac output as a result of structural and/or functional abnormality of the heart (1). The prevalence of HF increases with age, with an incidence rate of 1% for persons aged <55 years to >10% among those aged ≥70 years (2). There are several known causes of HF, with coronary artery disease and hypertension being the most common in Western and developed countries (3). The prognosis for HF has improved over the last decades, with a mortality rate reported of 67% within five years after diagnosis (4). Quality of life among persons with HF is often reduced, as symptoms such as shortness of breath and fatigue impair physical function and the capability to carry out social activities, with the risk of social isolation as a consequence (5).

Frailty is common among persons with HF, with an estimated prevalence of around 45% (6). Frailty is a clinical condition that increases with older age and is characterized by increased vulnerability to stress (7). Frail, older persons have an increased risk of adverse outcomes such as falls, disability, long-term care, and death (8). One important component in the frailty phenotype, as described by Fried et al. (9), is poor nutrition in terms of weight loss and sarcopenia, e.g., low muscle mass and decreased strength. In a recent review and meta-analysis, the prevalence of malnutrition among persons with HF was found to be 46%, and the all-cause mortality of those individuals with malnutrition was almost double compared with those without malnutrition (10). Another significant component of frailty is reduced functional capacity, which may impair quality of life. Munk et al. (11) reported that a nutritional intervention for older persons who had been discharged from the hospital improved both physical function and quality of life. Thus, effective interventions with the potential to treat malnutrition and improve frailty are important for persons with HF, as they may reduce mortality and increase functional capacity and quality of life.

Several underlying mechanisms for malnutrition, sarcopenia, and frailty among persons with HF have been reported. Reduced nutrient intake may be attributed to loss of appetite and lack of energy to prepare food (12). Furthermore, several metabolic abnormalities have been found in skeletal muscles in persons with HF, which may contribute to sarcopenia and loss of muscle mass. For example, compared to healthy persons, those with HF have a shortage of energy in muscle (13), reduced muscle blood flow and early anaerobic metabolism during exercise (14), as well as intrinsic changes in skeletal muscle fibres/histology and biochemistry (15). Older persons in general have been found to require higher doses of protein or amino acids to achieve muscle protein synthesis in comparison to younger persons (16). Overall, vitamin D deficiency has been associated with muscle weakness and sarcopenia among older persons (17, 18). Additionally, impaired bowel perfusion may lead to malabsorption and protein loss from the gut (19).

Previous research on nutritional interventions in HF has been performed with diverse outcomes and is summarised in a review by Billingsley et al (20). Sodium restriction and fluid restriction have been studied to reduce fluid retention, thereby achieving a reduction in HF signs and symptoms. The results conflicted with some studies reporting that sodium restriction improved the amount of extracellular fluid, fatigue, and quality of life. On the contrary, other studies found increased readmissions and hospitalizations among persons on sodium restriction and decreased energy intake. Dietary patterns such as the Mediterranean diet and Dietary Approaches to Stop Hypertension (DASH) have been reported to prevent the onset of HF and possibly reduce HF signs and symptoms and quality of life. Caloric restriction has been studied in overweight persons with HF (BMI ≥30), that has resulted in reduced body weight and improved glucose control and cardiac function (21). On the other hand, unintentional weight loss is a risk factor for malnutrition, which in turn is associated with increased mortality. A common strategy to increase weight and muscle mass in malnutrition is energy and/or protein supplementation (20).

The overall purpose of this review was to provide an overview of nutrition-related interventions to improve the components of frailty in older persons with HF by 1) identifying and describing nutrition interventions evaluated to improve nutritional and functional outcomes in older persons with HF and 2) summarising and describing the effects of these nutrition interventions.

## Methods

### Search strategies

To identify potentially relevant articles, the online databases Medline, Embase, Web of Science, and Cinahl were searched in June 2022. No time limit was applied. The final search strategies were drafted by an experienced librarian in collaboration with two of the authors (KB, NW) and included the search terms ‘heart failure’, ‘malnutrition’, and ‘frailty’. The complete search strategy is presented as a supplementary file.

Each record title and abstract were screened by two out of a total of three reviewers (KB, NW, GF) working independently in a blinded process facilitated by the Rayyan free tool for systematic reviews (22). After the screening, all three reviewers met to discuss inconsistencies in their judgment of records.

To find more relevant articles, the reference lists of articles identified through the database search mentioned above were screened, as were the reference lists of four previously published literature reviews regarding HF and nutrition (12, 20, 21, 23).

### Inclusion criteria

The search strategies were based on the following PICO:

- Population: Older persons (mean age ≥65 years) with chronic HF with no further specification.
- Intervention: Any nutritional intervention
- Control: No restrictions regarding the control group.
- Outcome: Measures related to frailty as defined by Fried et al (9), such as nutritional status (e.g., body composition, weight change) or functional measures (e.g., hand grip strength, timed get up and go test (TGUG), 6-minute walk test (6MWT)).

Original articles in the English language reporting any nutritional intervention evaluated in a group of older persons (mean age ≥65 years) with HF and outcomes related to the concept of frailty (9)—i.e., body composition/sarcopenia and functional capability—were included in the review. We included randomised controlled trials, controlled trials, and feasibility studies.

### Quality assessment

The Revised Cochrane risk-of-bias tool for randomised trials (24) was used to assess the methodological quality of the studies. The risk of bias was assessed by the first author (KB).

### Data synthesis and presentation

There were large variations among the articles regarding interventions and methodology, so a narrative review approach was chosen to present the results (25). The results are presented according to the nature of the intervention studied. Data were extracted by the first author (KB). The PRISMA checklist (26) was followed and is available as a supplementary file. No review protocol was prepared for this study.

## Results

### Selection process

After removing duplicates from the database search, the remaining 1083 records were screened, resulting in seven articles eligible for more thorough reading. Of these, only two articles were found to meet inclusion criteria for the review.

Screening the reference lists of the seven articles mentioned above resulted in an additional ten articles. Screening the reference lists of the four previously published literature reviews regarding HF and nutrition resulted in an additional 20 articles. These were retrieved and read thoroughly, of which six were found eligible for inclusion in the review. Thus, a total of eight articles were included in the final review.

**Figure 1.**
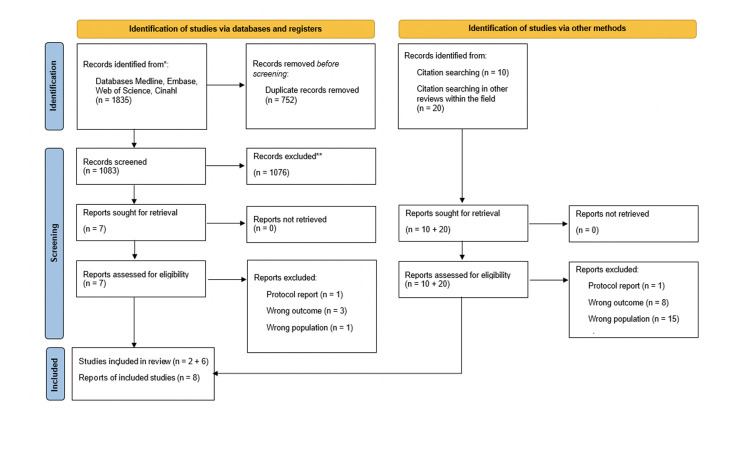
Prisma 2020 flow diagram of literature search

See Figure 1 for an overview of the process of identifying eligible articles.

### Study characteristics

**Figure 2.**
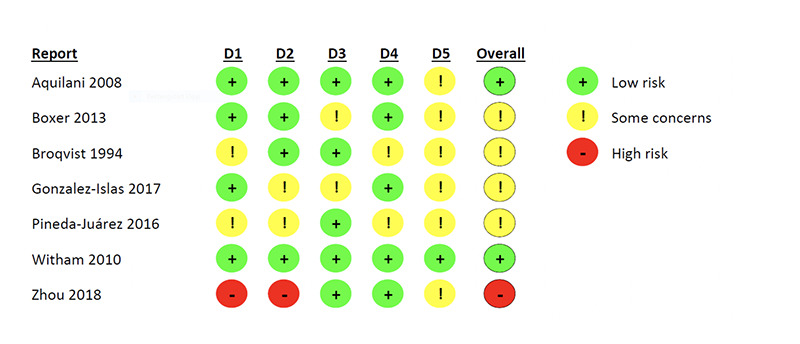
Risk of bias assessment using the Risk of Bias Assessment tool RoB2

Among the eight included studies, six were randomised controlled trials (RCT’s), one was a controlled trial, and one was a feasibility study. Table 1 shows the characteristics of the included studies. Year of publication varied between 1994 to 2017. The number of participants included in the RCTs fell between 22 (27) and 105 (28). Countries of origin included Italy (29), the United States (30, 31), Sweden (27), Mexico (32, 33), the United Kingdom (28), and China (34). Risk of bias was assessed in the controlled trials, and of these (n =7) two were judged to have low risk of bias, five had some concerns of bias, and one was assessed to be at high risk of bias. Figure 2 summarise the dimensions of the risk of bias assessment.

D1: Bias arising from the randomization process, D2: Bias due to deviations from intended interventions, D3: Bias due to missing outcome data, D4: bias in measurement of the outcome, D5: bias in selection of the reported result

**Table 1.** Data extraction from included studies

Author, year, country, setting, journal	Study design	Study population	Sample size (N) Gender (M/F) Age NYHA class	Intervention Duration of intervention	Control	Selected outcomes assessed	Results
Aquilani, 2008 Italy Outpatients selected from clinical databases European Journal of heart failure	Randomized, controlled study	Chronic BMI 20- 25 Arm muscle area <10 percentile Energy intake ≥30 kcal/kg/day and protein >1,1 g/kg Adequate daily physical activity	44 patients included. Dropouts: IG n=1, CG n=5 38 patients completed. IG n=21, CG n=17 Male n=27, female n=11 Age 74.5±5 (IG); 73.1±2.8 (CG) NYHA I n=0, NYHA II n=27, NYHA III n=11, NYHA IV n=0	Oral mixture supplement with 8 g essential amino acids (4 g twice daily). 2 months	No supplements.	1. Anthropometric evaluations (body weight, BMI, arm muscle area) 2. Exercise capacity (6MWT, bicycle exercise test) 3. Nitrogen balance 4. Nutritional intake (7 day food diary)	Plasma leucine increased in IG, suggesting good compliance. 1. Increase in body weight >1 kg was more frequent in IG (80% vs 30%). Muscle mass (arm muscle area) increased in both groups. 3. IG group improved exercise capacity, no difference in CG. 4. Nitrogen balance no difference. 5. No difference in nutritional (energy and protein) intake in CG and IG.
Boxer, 2013 United States Academic medical center JACC: Heart Failure	Parallel design, double-blind RCT	Age ≥50 years NYHA class II-IV Serum 25(OH) D level <37,5 ng/l.	64 patients included and completed. IG n=31, CG n=33 Male n=33, female n=31 Age 65.8±10.6 (IG); 66.0±10.4 (CG) NYHA III II n=41, NYHA n=29, NYHA IV n=0	Weekly vitamin D3 50,000 IU + Calcium citrate 400 mg twice a day 6 months	Placebo + Calcium citrate 400 mg twice a day	Functional evaluation (TGUG, 6MWT)	Significantly larger increase in mean serum 25 (OH) D levels in IG. No differences between groups in TGUG, 6MWT.
Broqvist, 1994 Sweden Setting not specified European Heart Journal	Randomized double-blind controlled trial	NYHA III-IV	22 patients included and completed. IG n=9, CG n=13 Male n=19, female n=3 Age 70±2 (IG); 73±2 (CG)	500 ml ONS (30 g protein, 750 kcal), ingested between meals in addition to normal food intake. Duration of 8 weeks.	500 ml 1:10 diluted placebo version	1. Malnutrition signs incl anthropometric (weight index, MAC, TSF, AMC) and laboratory measures (blood samples). 2. Nutrient intake and energy balance 3. Exercise tolerance (peak oxygen uptake etc)	1. IG had a larger TSF compared to CG. No differences between groups regarding other signs e.g. transthyretin or albumin. 2. Increase in energy, fat and protein intake in IG. No change in intake from food. 3. No differences regarding exercise tolerance either between groups or in IG during the study.
George, 2017 United States Medical University hospital Journal of Physiotherapy & Rehabilitation	Feasibility study	Age>55 BMI 19-30 NYHA II-III	11 patients included, 6 complete IG: n=3, CG: n=3 Male n=3, female n=3 Age 84±0.58 (IG), 75±3.79 (CG)	Exercise instructions on DVD or pamphlet. Target 20 min 6 days/week (aerobic and resistance). Protein whey isolate powder in addition to normal diet, to reach 1,5 gram/kg/day. Duration 6 months	Usual care from primary care physician	Physical functioning (TGUG, hand grip strength, leg extension, 6MWT)	No significant differences between groups on physical function.
Gonzalez-Islas 2017 Mexico Heart failure clinic Nutrición Hospitalaria	Randomized controlled trial	NYHA class I-III ≥18 years old	88 patients included, 15 dropouts (lost to follow-up) 73 completed, IG: n=38, CG: n=35 Male n=35, female n=38 Age 68.47±12.65 (IG), 70.45±12.35(CG) NYHA I: II: n=19, n= 47, NYHA NYHA III: n=4	Low-carbohydrate group (40% carbohydrates, 20% protein, 40% fats of which 12% saturated fats) Normocaloric diets. Instructions in nutritional handbook and individual oral instructions. Duration 2 months	Standard diet according to American Heart Association Dietary guidelines (50% carbohydrates, 20% protein, 30% fats of which 10% saturated fats) Normocaloric diets.	1. Oxygen saturation 2. Body composition (weight, bioimpedance analysis) 3. Handgrip strength	IG reported decrease in intake of carbohydrates at end of study (statistically significant). No statistically significant differences between groups regarding energy, protein, fats, fiber or sodium. 1. Increase in oxygen saturation in IG, decrease in CG. 2. Weight loss in IG (NS). No differences in body composition between groups. 3. No statistical difference in hand grip strength
Pineda-Juarez, 2016 Mexico Heart failure clinic Clinical Nutrition	Randomized controlled trial	>18 years old Stable HF	N=66 patients included, 11 dropouts 55 completed, IG: n=29, CG: n=26 Male n=39, female n=27 Age 74,5 (61,7-80,2) (IG), 71 (58-79) (CG) NYHA I: n=39, NYHA n=20, NYHA III: n=4, NYHA IV: n=1	Resistance exercise program, 1 h, twice a week BCAA 10 g/day (5 g after breakfast, 5 g before exercise). 10 g protein subtracted from usual diet. All participants: individualized and standardized diet 50% carbohydrates, 20% protein, 30% fats. 4 meals/day, sodium 2400-3000 mg/day, liquids 2- 3 l/day. Duration: 12 weeks	Resistance exercise program, 1 hour twice a week All participants: individualized and standardized diet 50% carbohydrates, 20% protein, 30% fats. 4 meals/day, sodium 2400-3000 mg/day, liquids 2-3 l/day.	1. Anthropometric measurements (weight, arm circumference, waist, and hip). Handgrip strength. 2.Body composition (bioelectrical impedance analysis) 3. Diet measurement (24 h recall)	1. Changes in anthropometrical measurements by decrease in hip circumference, arm and waist, in both groups. Decrease in hip circumference statistically significantly larger in IG. Improvements in muscle strength in both groups. 2. Decrease in third space water in both groups (NS) 3. No significant changes between groups regarding diet.
Witham, 2010 United Kingdom Primary and secondary care Circulation: Heart Failure	Double-blind, randomized, placebo-controlled trial	≥70 years old NYHA-class II-III 25 hydroxyvitamin D level <50 mmol/L	N=105 patients included, 9 dropouts IG: n=53, CG: n=52 Male n=69, Female n=36 Age 78.8±5.6 (IG), 80.6± 5.7 (CG) NYHA II: n=47, NYHA III: N=58	One oral dose 100 000 units ergocalciferol at baseline and after 10 weeks	Placebo	1. 6MWT 2. TGUG 3. Daily physical activity (accelerometer 7 days)	25-hydroxyvitamin D levels increased in IG. 1. No significant improvement in 6MWT in IG. 2. No improvement in IG 3. No improvement in IG
Zhou, 2018 China Hospital Clinical interventions in aging	Controlled trial	≥ 65 years old At nutritional risk (NRS >3) Hospitalized with heart failure	N= 105 patients IG A: n= 35, IG B: n=35, CG: n=35 Male n=63, female n=42 Age 71 (65-82) NYHA I: n=28, NYHA II: n=42, NYHA III: n=35	IG Group A: nasal tube feeding 500 ml for 1 month. IG Group B: 5 nasal tube feeding 500 ml for 3 months. Enteral nutrition 450 kcal, 17 g protein	CG: free diet (controlled amount of sodium)	1. Anthropometrical measures: BMI, TSF, AMC	1. Statistically significant improvements in BMI, TSF, AMC. These parameters increased in CG also, but not statistically significant.

6MWT six-minute walk test, AMC arm muscle circumference, BCAA branched chain amino acids, BMI body mass index, CG control group, HF heart failure, IG circumference, ONS oral nutritional supplement, NRS nutritional risk screening, NYHA New York Heart Association Classification, NS not statistically significant, intervention group, MAC mid-arm ONS oral nutritional supplement, TGUG timed get up and go, TSF triceps skin fold

### Participants and interventions

The severity of chronic HF among participants in the included studies varied regarding NYHA score (see Table 1). The mean age of participants ranged from 65 (30) to 84 years old (31). Depending on intervention and outcome, some studies had additional inclusion criteria, such as low muscle mass (29), low levels of serum 25-hydroxyvitamin D (28, 30), BMI within a specific range (31), or being at nutritional risk (34).

The interventions all aimed at improving components of frailty, such as body composition or nutritional or functional performance, but took different approaches. Thus, in the presentation of the results, studies were grouped according to the type of intervention used. Three studies hypothesised that supplementing protein or amino acids would improve nutritional status and protein synthesis (29, 31, 33). Two studies used supplements, including energy, protein, and other nutrients, in order to improve nutritional status (27, 34). Two studies investigated the effects of vitamin D on functional performance (28, 30). One study investigated the effects of a low carbohydrate diet on body composition (32).

### Summary of interventions and results

#### Supplementation of protein or amino acids

Three studies investigated the supplementation of protein or amino acids on improving nutritional status through increased weight and muscle mass (29, 33) or functional performance (31). The rationale for this among all three studies was to improve muscle synthesis by addressing anabolic resistance. The studies using amino acids instead of whole protein (29, 33) referred to amino acids as being more effective in the aforementioned goals by enhancing protein turnover and muscle metabolism. George et al. (31) and Pineda Suarez et al. (33) combined protein supplementation or branched-chain amino acids (BCAA) with exercise, whereas Aquilani et al. (29) did not combine supplementation of essential amino acids with any physical activity intervention; however, one inclusion criteria was that study participants already had adequate daily physical activity.

The amount of protein supplementation was either flexible, to reach a general goal of total protein intake (1.5 grams protein per kilogram body weight/day in George et al. (31)) or as a supplement of 8–10 grams of amino acids a day to the usual diet. However, Pineda Suarez et al. subtracted the 10 grams of (BCAA) from the total protein intake in order to reach a total protein intake of 20% in relation to fats and carbohydrates.

The duration of the interventions varied by two months (29), 12 weeks (33), and six months (31). Control groups received exercise programs without protein supplementation (33), usual care (31), or no supplementation (29).

The three papers had conflicting results. The study by Aquilani et al. (29) reported that participants in the intervention group who received essential amino acids improved 6MWT and had a larger increase in weight compared to the control group. This study was judged to have a low risk of bias.

In contrast, Pineda Suarez et al. (33) reported improvements in hand grip strength in both intervention and control groups and therefore concluded that these improvements resulted from the exercise intervention independent of the BCAA supplementation. This study was found to have some concerns regarding the methodology, which included uncertainty of concealment of allocation sequence at randomisation, and it was unclear whether outcome assessors were aware of whether the participant had been in the control or the intervention group.

George et al. (31) was a feasibility study with only 11 participants included (of whom 6 completed the study). They found no statistically significant difference regarding functional performance measured by TGUG, hand grip strength, and 6MWT.

#### Supplementation of energy, protein, and other nutrients

Two studies focused on nutritional support through liquid supplementation, including energy, protein, and other nutrients in order to improve nutritional status (27, 34). Broqvist et al. (27) used oral nutritional supplements, providing a daily intake of 750kcal and 30g of protein in addition to the usual dietary intake. Zhou et al. (34) provided 500mL supplementation of enteral nutrition (EN) through nasogastric tube feeding, containing 450kcal and 17g protein (product name: RuiDai; Fresenius Kabi Deutschland GmbH, 500 ml/bottle). The interventions lasted eight weeks (27) and either one or three months, as Zhou et al. (34) had two intervention groups with different intervention times. The control group in Broqvist et al. (27) received a diluted version of the oral nutritional supplement, whereas Zhou et al. (34) had a control group with no nutritional support.

Both studies found improvements in nutritional status after the intervention in the form of increased muscle and fat mass, measured through arm muscle circumference and triceps skin fold. However, it is important to note that these results should be interpreted with caution, as the study by Zhou had several methodological concerns, including no randomisation and a lack of blinding.

#### Supplementation of Vitamin D

Two studies investigated vitamin D supplementation to improve functional performance (28, 30). The rationale behind this approach was that low levels of 25-hydroxyvitamin D is common among older persons with HF. In addition, vitamin D deficiency also has been associated with low functional capacity and skeletal myopathy. Thus, the authors hypothesised that improving vitamin D status among those with vitamin D deficiency would, in turn, improve physical capacity. None of the studies combined the vitamin D supplement with any exercise intervention. Control groups received a placebo.

The studies lasted 20 weeks (28) and six months (30). The administration of the vitamin D supplement equaled a daily vitamin D intake of 35μg/1428 IU (28) or 179μg/7143 IU (30).

Both studies reported good compliance with vitamin D supplementation and were judged to be of good quality in the risk of bias assessment. None of the studies found any improvement in the functional measures of TGUG and 6MWT.

#### Diet approach—low carbohydrate diet

One study investigated the effect of a low-carbohydrate diet on oxygen saturation, body composition, and clinical variables in persons with HF (32). The rationale behind the design was that HF compromises respiratory efficiency through impaired oxygen consumption, and in other populations, a diet with limited energy contribution from carbohydrates and an increase in fat has been associated with better respiratory efficiency.

The study diet consisted of 40% carbohydrates, 20% protein, and 40% fats (12% saturated fats). The control group was recommended a standard diet according to American Heart Association Dietary guidelines (50% carbohydrates and 30% fats). Both the intervention and control diet were normocaloric. The duration of the study was two months. By the end of the study, no statistically significant differences regarding body composition measured with bioimpedance analysis and hand grip strength were found. However, the authors reported a statistically significant increase in oxygen saturation in the intervention group. There were some concerns regarding risk of bias in this study, as participants and personnel delivering the intervention were naturally not blinded and drop-outs in the study were younger than those who completed.

## Discussion

Here, we have summarised current evidence on nutritional interventions to improve the frailty of older persons with heart failure. As described in the introduction, previous research has focused on sodium restriction, dietary patterns, or caloric restriction. However, these kinds of interventions are often not appropriate in persons with frailty. Because frailty often includes malnutrition and/or sarcopenia, there is a risk of reduced energy and nutrient intake with restrictive diets. Therefore, it is unsurprising that most of the included studies had interventions that added to the study participants’ diets rather than restricted them, with one exception in the study by Gonzalez-Islas (32). In contrast, five of the eight papers studied macronutrients such as protein supplementation with or without extra energy. Two papers investigated the effect of high vitamin D supplementation.

## Results discussion

### Supplementation with protein or amino acids

This review supports the evidence that protein and physical exercise are needed to build muscle mass. The studies using protein supplementation found improvements in outcomes. One of the studies (33) combined protein supplementation (BCAA) with exercise, compared with exercise alone in the control group. As both groups improved muscle strength, the authors referred the positive result to the exercise part of the intervention. On the other hand, the study by Aquilani et al. (29) reported that protein supplementation (essential amino acids) among persons with adequate physical activity improved nutritional status. However, it should be noted that the amount of protein supplementation was rather modest (8–10 grams) while it has been suggested that older persons would require up to 40 grams of extra protein to stimulate protein synthesis (35). Previous research has shown that protein supplementation alone may not be sufficient to achieve a change in muscle strength, but that exercise is necessary (36).

### Vitamin D

Previous research has established the association between vitamin D and lower muscle function—e.g., hand grip strength (37). Despite this, none of the studies included here found any positive effects on functional measures, which is in line with a systematic review of two large interventions on vitamin D supplementation for older persons (38).

### Combination of energy, protein, and other nutrients

One of the two included studies used oral nutritional supplements (ONS) and the other used enteral nutrition (EN) through a nasogastric tube. The use of ONS in older persons with malnutrition or risk of malnutrition is a Grade A recommendation by the European Society of Enteral and Parenteral Nutrition (ESPEN) (7). Several studies have shown that ONS, as a supplement to ordinary food intake, improves dietary intake and body weight and lowers the risk of complications (39) during hospital care and functional decline after discharge (40). This is in line with the result from the study included in this review, where the authors found increased weight and triceps skin fold measurements (27). Zhou et al. (34) provided the EN supplement through a nasogastric tube; however, the rationale behind this administration method is not specified in the article. The ESPEN recommendation is to initiate EN when oral intake is expected to be impossible for more than three days or <50% of estimated energy needs more than one week despite interventions to optimise oral intake (7). Such a situation could be dysphagia or acute illness. Thus, the practical implementation of the intervention described is probably not feasible in clinical practice. On the other hand, Zhou et al. (34) demonstrate that the addition of supplemental energy and nutrients may improve nutritional status and that this improvement also increases over time.

### Low carbohydrate diet

Finally, the study that used a low carbohydrate diet reported no statistically significant differences in hand grip strength after two months (32). However, they did report a statistically significant improvement in oxygen saturation in the intervention group. The association between a diet low in carbohydrates and higher in fat and reduced respiratory effort has been studied in persons with obstructive pulmonary disease and was recently summarised in a systematic review by Guerra et al. (41). They did report an association between ventilatory parameters and a higher intake of fat relative to carbohydrates. Still, they reported that the possible clinical advantages for the individual had not been investigated. Thus, they concluded that the evidence grade was not strong enough to come to any recommendations. The low-carbohydrate diet investigated by Gonzalez-Islas et al. (32) was rather similar to the Mediterranean diet, defined as a distribution of macronutrients equating to 37% total fat (9% saturated fat), 15% protein and 43% carbohydrates (42). The Mediterranean diet has been extensively studied with coronary heart disease and specifically in preventing or improving clinical outcomes in HF (43). The results have been conflicting, and current HF recommendations from the European Society of Cardiology do not specify dietary advice other than striving for a healthy diet and preventing malnutrition (1).

## Methodological considerations

One of this study’s strengths is the focus on nutritional and functional outcomes related to frailty, which may be more relevant for the individual’s everyday life and function than clinically oriented outcomes such as mortality. Another strength is the systematic methodology for identifying articles and the risk of bias assessment.

This study also has limitations. There is a large variation among interventions and outcomes in the included studies, which does not allow for meta-analyses or pooling of data. Several studies are carried out in small populations, and 4 out of 8 were published ≥10 years ago. Not all studies had frailty as inclusion criteria, and thus, the generalisability of the results to this population must be made with caution.

## Conclusion and further research

The results from this review suggest that components of frailty, such as muscle mass and functional capacity, can be improved with energy and protein supplementation, especially in combination with physical exercise. This highlights the importance of nutritional and exercise interventions as an integrated part of clinical care of heart failure in older persons. Further research is needed to find out which combination of energy, protein, or amino acids is the most efficient and how this should be combined with physical exercise.

## Documentation of search strategies University Library search consultation group

Additional material Supplementary file supplied by authors.

Date: June 2022

Topic/research question: Malnutrition in frail persons with heart failure

Name of researcher(s): Kerstin Belqaid

Librarian(s): Love Strandberg, Emma-Lotta Säätelä

Databases:

1. Medline (Ovid)
2. Embase (embase.com)
3. Web of Science (Clarivate)
4. Cinahl (Ebsco)

Total number of hits:

- Before deduplication: 1,835
- After deduplication: 1,083

Comments:

Deduplication based on the method described in: Bramer, W. M., Giustini, D., de Jonge, G. B., Holland, L., & Bekhuis, T. (2016). De-duplication of database search results for systematic reviews in EndNote. *Journal of the Medical Library Association: JMLA*, 104(3), 240–243. doi:10.3163/1536-5050.104.3.014

One final, extra step was added to compare DOIs.

**Table:** 1. Medline

Interface: Ovid MEDLINE(R) and Epub Ahead of Print, In-Process & Other Non-Indexed Citations and Daily Date of Search: 21 June 2022 Number of hits: 395 Comment: In Ovid, two or more words are automatically searched as phrases; therefore no quotation marks are needed	Field labels exp/ = exploded MeSH term / = non exploded MeSH term .ti,ab,kf. = title, abstract and author keywords .hw = word in MeSH-term adjx = within x words, regardless of order * = truncation of word for alternate endings

**#**	**Search query**	**Hits**
1	exp Heart Failure/	139141
2	exp Heart Arrest/	53950
3	Cardiomyopathy, Dilated/	16746
4	Shock, Cardiogenic/	10102
5	exp Ventricular Dysfunction/	41664
6	Cardiac Output, Low/	5579
7	((cardiac or cardiocirculatory or cardio-circulatory or cardiogenic or cardiomyopath* or cardiopulmonary or cardiovascular or heart or myocardial or ventricle* or ventricular) adj3 (arrest or collapse or failure* or insufficienc* or decompensat* or shock?)).ti,ab,kf.	288084
8	((diastolic or systolic or ventricle? or ventricular) adj3 (dysfunction or overload)).ti,ab,kf.	41958
9	(heart edema? or heart oedema?).ti,ab,kf.	28
10	((cardiorenal or cardio-renal) adj3 syndrome).ti,ab,kf.	1522
11	or/1-10	395516
12	(deficienc* or diet* or eat* or energy or fats or fatty or nutrient* or nutrition* or protein*).hw.	4378067
13	exp Malnutrition/	131441
14	exp Food/	1410641
15	(calorie* or deficienc* or diet* or eat* or energy or fats or fatty or food or malnutri* or malnourish* or nutrient* or nutrition* or protein* or undernutri* or undernourish*).ti,ab,kf.	5541659
16	or/12-15	7832574
17	Frailty/	6875
18	Frail Elderly/	13998
19	(fragility or frail* or debilit* or functionally impair*).ti,ab,kf.	81449
20	((SOF or CHS) adj3 index*).ti,ab,kf.	59
21	or/17-20	85828
22	11 and 16 and 21	395

**Table:** 2. Embase

Interface: embase.com Date of Search: 21 June 2022 Number of hits: 799 Comment: Emtree is the controlled vocabulary in Embase	Field labels /exp = exploded Emtree term /de = non exploded Emtree term ti,ab,kw = title, abstract and author keywords NEAR/x = within x words, regardless of order * = truncation of word for alternate endings

**No.**	**Query**	**Results**
#1	'heart failure'/exp	601824
#2	'congestive cardiomyopathy'/exp	35723
#3	((cardiac OR cardiocirculatory OR 'cardio circulatory' OR cardiogenic OR cardiomyopath* OR cardiopulmonary OR cardiovascular OR heart OR myocardial OR ventricle* OR ventricular) NEAR/3 (arrest OR collapse OR failure* OR insufficienc* OR decompensat* OR shock?)):ti,ab,kw	449782
#4	((diastolic OR systolic OR ventricle? OR ventricular) NEAR/3 (dysfunction OR overload)):ti,ab,kw	73541
#5	'heart edema*':ti,ab,kw OR 'heart oedema*':ti,ab,kw	41
#6	((cardiorenal OR 'cardio renal') NEAR/3 syndrome):ti,ab,kw	2687
#7	#1 OR #2 OR #3 OR #4 OR #5 OR #6	724706
#8	'nutrition'/exp	2567327
#9	'malnutrition'/exp	193043
#10	'nutritional deficiency'/exp	301996
#11	calorie*:ti,ab,kw OR deficienc*:ti,ab,kw OR diet*:ti,ab,kw OR eat*:ti,ab,kw OR energy:ti,ab,kw OR fats:ti,ab,kw OR fatty:ti,ab,kw OR food:ti,ab,kw OR malnutri*:ti,ab,kw OR malnourish*:ti,ab,kw OR nutrient*:ti,ab,kw OR nutrition*:ti,ab,kw OR protein*:ti,ab,kw OR undernutri*:ti,ab,kw OR undernourish*:ti,ab,kw	6652494
#12	#8 OR #9 OR #10	8389479
#13	'frailty'/de	19947
#14	'frail elderly'/de	11522
#15	fragility:ti,ab,kw OR frail*:ti,ab,kw OR debilit*:ti,ab,kw OR 'functionally impair*':ti,ab,kw	120528
#16	((sof OR chs) NEAR/3 index*):ti,ab,kw	105
#17	#12 OR #13 OR #14 OR #15	126136
#18	#7 AND #12 AND #17	1162
#19	#18 NOT ('conference abstract'/it OR 'conference paper'/it OR 'conference review'/it)	799

**Table:** 3. Web of Science Core Collection

Interface: Clarivate Analytics Editions = A&HCI , ESCI , SCI-EXPANDED , SSCI Date of Search: 22 June 2022 Number of hits: 488	Field labels TS/Topic = title, abstract, author keywords and Keywords Plus NEAR/x = within x words, regardless of order * = truncation of word for alternate endings Note: the *Exact search*-function was used for all the searches

**#**	**Query**	**Hits**
1	TS=((cardiac or cardiocirculatory or cardio-circulatory or cardiogenic or cardiomyopath* or cardiopulmonary or cardiovascular or heart or myocardial or ventricle* or ventricular) NEAR/2 (arrest or collapse or failure* or insufficienc* or decompensat* or shock?))	350,165
2	TS=((diastolic or systolic or ventricle? or ventricular) NEAR/2 (dysfunction or overload))	55,714
3	TS=(heart edema? or heart oedema?)	72
4	TS=((cardiorenal or cardio-renal) NEAR/2 syndrome)	1,998
5	#4 OR #3 OR #2 OR #1	378,566
6	TS=(calorie* or deficienc* or diet* or eat* or energy or fats or fatty or food or malnutri* or malnourish* or nutrient* or nutrition* or protein* or undernutri* or undernourish*)	9,611,046
7	TS=(fragility or frail* or debilit* or "functionally impair*")	99,409
8	TS=((SOF or CHS) NEAR/2 index*)	62
9	#8 OR #7	99,424
10	#9 AND #6 AND #5	488

**Table:** 6. Cinahl

Interface: Ebsco Date of Search: 22 June 2022 Number of hits: 153	Field labels MH+ = exploded Cinahl Heading MH = non exploded Cinahl Heading MW = word in Cinahl Heading TI = title AB = abstract Nx = within x words, regardless of order * = truncation of word for alternate endings

#	Query	Results
S1	(MH "Heart Failure+")	47,317
S2	TI ( ((cardiac OR cardiocirculatory OR "cardio-circulatory" OR cardiogenic OR cardiomyopath* OR cardiopulmonary OR cardiovascular OR heart OR myocardial OR ventricle* OR ventricular) N3 (arrest OR collapse OR failure* OR insufficienc* OR decompensat* OR shock?)) ) OR AB ( ((cardiac OR cardiocirculatory OR "cardio-circulatory" OR cardiogenic OR cardiomyopath* OR cardiopulmonary OR cardiovascular OR heart OR myocardial OR ventricle* OR ventricular) N3 (arrest OR collapse OR failure* OR insufficienc* OR decompensat* OR shock?)) )	88,187
S3	TI ( ((diastolic OR systolic OR ventricle? OR ventricular) N3 (dysfunction OR overload)) ) OR AB ( ((diastolic OR systolic OR ventricle? OR ventricular) N3 (dysfunction OR overload)) )	10,774
S4	TI ( "heart edema*" OR "heart oedema*" ) OR AB ( "heart edema*" OR "heart oedema*" )	0
S5	TI ( ((cardiorenal or cardio-renal) N3 syndrome) ) OR AB ( ((cardiorenal or cardio-renal) N3 syndrome) )	354
S6	S1 OR S2 OR S3 OR S4 OR S5	106,766
S7	MW (deficienc* or diet* or eat* or energy or fats or fatty or nutrient* or nutrition* or protein*)	511,985
S8	(MH "Nutrition+")	179,712
S9	(MH "Nutrition disorders+")	147,993
S10	(MH "Food+")	195,776
S11	TI ( (calorie* or deficienc* or diet* or eat* or energy or fats or fatty or food or malnutri* or malnourish* or nutrient* or nutrition* or protein* or undernutri* or undernourish*) ) OR AB ( (calorie* or deficienc* or diet* or eat* or energy or fats or fatty or food or malnutri* or malnourish* or nutrient* or nutrition* or protein* or undernutri* or undernourish*) )	622,296
S12	S7 OR S8 OR S9 OR S10 OR S11	825,755
S13	(MH "Frail Elderly")	8,431
S14	(MH "Frailty syndrome")	3,507
S15	TI ( (fragility or frail* or debilit* or "functionally impair*") ) OR AB ( (fragility or frail* or debilit* or "functionally impair*") )	28,920
S16	S13 OR S14 OR S15	32,327
S17	S6 AND S12 AND S16	153

## PRISMA 2020 Checklist

Additional material Supplementary file supplied by authors.

**Table:** 

Section and Topic	Item #	Checklist item	Location where item is reported
**TITLE**			
Title	1	Identify the report as a systematic review.	Title
**ABSTRACT**			
Abstract	2	See the PRISMA 2020 for Abstracts checklist.	Abstract
**INTRODUCTION**			
Rationale	3	Describe the rationale for the review in the context of existing knowledge.	Introduction §4-5
Objectives	4	Provide an explicit statement of the objective(s) or question(s) the review addresses.	Introduction §5
**METHODS**			
Eligibility criteria	5	Specify the inclusion and exclusion criteria for the review and how studies were grouped for the syntheses.	Inclusion criteria
Information sources	6	Specify all databases, registers, websites, organisations, reference lists and other sources searched or consulted to identify studies. Specify the date when each source was last searched or consulted.	Search strategies, Suppl. file
Search strategy	7	Present the full search strategies for all databases, registers and websites, including any filters and limits used.	Suppl. file
Selection process	8	Specify the methods used to decide whether a study met the inclusion criteria of the review, including how many reviewers screened each record and each report retrieved, whether they worked independently, and if applicable, details of automation tools used in the process.	Search strategies
Data collection process	9	Specify the methods used to collect data from reports, including how many reviewers collected data from each report, whether they worked independently, any processes for obtaining or confirming data from study investigators, and if applicable, details of automation tools used in the process.	Data synthesis and presentation
Data items	10a	List and define all outcomes for which data were sought. Specify whether all results that were compatible with each outcome domain in each study were sought (e.g. for all measures, time points, analyses), and if not, the methods used to decide which results to collect.	Table 1
	10b	List and define all other variables for which data were sought (e.g. participant and intervention characteristics, funding sources). Describe any assumptions made about any missing or unclear information.	Table 1
Study risk of bias assessment	11	Specify the methods used to assess risk of bias in the included studies, including details of the tool(s) used, how many reviewers assessed each study and whether they worked independently, and if applicable, details of automation tools used in the process.	Quality assessment
Effect measures	12	Specify for each outcome the effect measure(s) (e.g. risk ratio, mean difference) used in the synthesis or presentation of results.	NA
Synthesis methods	13a	Describe the processes used to decide which studies were eligible for each synthesis (e.g. tabulating the study intervention characteristics and comparing against the planned groups for each synthesis (item #5)).	NA
	13b	Describe any methods required to prepare the data for presentation or synthesis, such as handling of missing summary statistics, or data conversions.	NA
	13c	Describe any methods used to tabulate or visually display results of individual studies and syntheses.	NA
	13d	Describe any methods used to synthesize results and provide a rationale for the choice(s). If meta-analysis was performed, describe the model(s), method(s) to identify the presence and extent of statistical heterogeneity, and software package(s) used.	Data synthesis and presentation
	13e	Describe any methods used to explore possible causes of heterogeneity among study results (e.g. subgroup analysis, meta-regression).	NA
	13f	Describe any sensitivity analyses conducted to assess robustness of the synthesized results.	NA
Reporting bias assessment	14	Describe any methods used to assess risk of bias due to missing results in a synthesis (arising from reporting biases).	Quality assessment
Certainty assessment	15	Describe any methods used to assess certainty (or confidence) in the body of evidence for an outcome.	NA
**RESULTS**			
Study selection	16a	Describe the results of the search and selection process, from the number of records identified in the search to the number of studies included in the review, ideally using a flow diagram.	Figure 1
	16b	Cite studies that might appear to meet the inclusion criteria, but which were excluded, and explain why they were excluded.	NA
Study characteristics	17	Cite each included study and present its characteristics.	Study characteristics, Table 1
Risk of bias in studies	18	Present assessments of risk of bias for each included study.	Figure 2
Results of individual studies	19	For all outcomes, present, for each study: (a) summary statistics for each group (where appropriate) and (b) an effect estimate and its precision (e.g. confidence/credible interval), ideally using structured tables or plots.	NA
Results of syntheses	20a	For each synthesis, briefly summarise the characteristics and risk of bias among contributing studies.	Summary of interventions and results
	20b	Present results of all statistical syntheses conducted. If meta-analysis was done, present for each the summary estimate and its precision (e.g. confidence/credible interval) and measures of statistical heterogeneity. If comparing groups, describe the direction of the effect.	NA
	20c	Present results of all investigations of possible causes of heterogeneity among study results.	NA
	20d	Present results of all sensitivity analyses conducted to assess the robustness of the synthesized results.	NA
Reporting biases	21	Present assessments of risk of bias due to missing results (arising from reporting biases) for each synthesis assessed.	NA
Certainty of evidence	22	Present assessments of certainty (or confidence) in the body of evidence for each outcome assessed.	NA
**DISCUSSION**			
Discussion	23a	Provide a general interpretation of the results in the context of other evidence.	Results discussion
	23b	Discuss any limitations of the evidence included in the review.	Methodological considerations
	23c	Discuss any limitations of the review processes used.	Methodological considerations
	23d	Discuss implications of the results for practice, policy, and future research.	Conclusions and further research
**OTHER INFORMATION**			
Registration and protocol	24a	Provide registration information for the review, including register name and registration number, or state that the review was not registered.	Data synthesis and presentation
	24b	Indicate where the review protocol can be accessed, or state that a protocol was not prepared.	Data synthesis and presentation
	24c	Describe and explain any amendments to information provided at registration or in the protocol.	NA
Support	25	Describe sources of financial or non-financial support for the review, and the role of the funders or sponsors in the review.	Funding
Competing interests	26	Declare any competing interests of review authors.	Conflict of interests
Availability of data, code and other materials	27	Report which of the following are publicly available and where they can be found: template data collection forms; data extracted from included studies; data used for all analyses; analytic code; any other materials used in the review.	NA

*From:* Page MJ, McKenzie JE, Bossuyt PM, Boutron I, Hoffmann TC, Mulrow CD, et al. The PRISMA 2020 statement: an updated guideline for reporting systematic reviews. BMJ 2021;372:n71. doi: 10.1136/bmj.n71
