# Supplementary material for: Nutritional Interventions in Older, Frail Persons with Heart Failure—A Systematic Narrative Review
Source: JAR Life. 2024 Nov 5;13:99–107. doi: 10.14283/jarlife.2024.15 (PMC11549435; doi:10.14283/jarlife.2024.15)
Supplement: Additional material — Supplementary file supplied by authors. [file jarlife-13-0015-S1.docx]

Documentation of search strategies

University Library search consultation group

Date: June 2022

Topic/research question: Malnutrition in frail persons with heart failure

Name of researcher(s): Kerstin Belqaid

Librarian(s): Love Strandberg, Emma-Lotta Säätelä

Databases:

1. Medline (Ovid)
2. Embase (embase.com)
3. Web of Science (Clarivate)
4. Cinahl (Ebsco)

Total number of hits:

- Before deduplication: 1,835
- After deduplication: 1,083

Comments:

Deduplication based on the method described in:
Bramer, W. M., Giustini, D., de Jonge, G. B., Holland, L., & Bekhuis, T. (2016). De-duplication of database search results for systematic reviews in EndNote. *Journal of the Medical Library Association: JMLA*, 104(3), 240–243. doi:10.3163/1536-5050.104.3.014

One final, extra step was added to compare DOIs.

1. Medline

| Interface: Ovid MEDLINE(R) and Epub Ahead of Print, In-Process & Other Non-Indexed Citations and Daily  Date of Search: 21 June 2022  Number of hits: 395  Comment: In Ovid, two or more words are automatically searched as phrases; therefore no quotation marks are needed | Field labels   - exp/ = exploded MeSH term - / = non exploded MeSH term - .ti,ab,kf. = title, abstract and author keywords - .hw = word in MeSH-term - adjx = within x words, regardless of order - * = truncation of word for alternate endings |
| --- | --- |
| \| **#** \| **Search query** \| **Hits** \| \| --- \| --- \| --- \| \| 1 \| exp Heart Failure/ \| 139141 \| \| 2 \| exp Heart Arrest/ \| 53950 \| \| 3 \| Cardiomyopathy, Dilated/ \| 16746 \| \| 4 \| Shock, Cardiogenic/ \| 10102 \| \| 5 \| exp Ventricular Dysfunction/ \| 41664 \| \| 6 \| Cardiac Output, Low/ \| 5579 \| \| 7 \| ((cardiac or cardiocirculatory or cardio-circulatory or cardiogenic or cardiomyopath* or cardiopulmonary or cardiovascular or heart or myocardial or ventricle* or ventricular) adj3 (arrest or collapse or failure* or insufficienc* or decompensat* or shock?)).ti,ab,kf. \| 288084 \| \| 8 \| ((diastolic or systolic or ventricle? or ventricular) adj3 (dysfunction or overload)).ti,ab,kf. \| 41958 \| \| 9 \| (heart edema? or heart oedema?).ti,ab,kf. \| 28 \| \| 10 \| ((cardiorenal or cardio-renal) adj3 syndrome).ti,ab,kf. \| 1522 \| \| 11 \| or/1-10 \| 395516 \| \| 12 \| (deficienc* or diet* or eat* or energy or fats or fatty or nutrient* or nutrition* or protein*).hw. \| 4378067 \| \| 13 \| exp Malnutrition/ \| 131441 \| \| 14 \| exp Food/ \| 1410641 \| \| 15 \| (calorie* or deficienc* or diet* or eat* or energy or fats or fatty or food or malnutri* or malnourish* or nutrient* or nutrition* or protein* or undernutri* or undernourish*).ti,ab,kf. \| 5541659 \| \| 16 \| or/12-15 \| 7832574 \| \| 17 \| Frailty/ \| 6875 \| \| 18 \| Frail Elderly/ \| 13998 \| \| 19 \| (fragility or frail* or debilit* or functionally impair*).ti,ab,kf. \| 81449 \| \| 20 \| ((SOF or CHS) adj3 index*).ti,ab,kf. \| 59 \| \| 21 \| or/17-20 \| 85828 \| \| 22 \| 11 and 16 and 21 \| 395 \| | |

2. Embase

| Interface: embase.com  Date of Search: 21 June 2022  Number of hits: 799  Comment: Emtree is the controlled vocabulary in Embase | Field labels   - /exp = exploded Emtree term - /de = non exploded Emtree term - ti,ab,kw = title, abstract and author keywords - NEAR/x = within x words, regardless of order - * = truncation of word for alternate endings |
| --- | --- |
| \| **No.** \| **Query** \| **Results** \| \| --- \| --- \| --- \| \| #1 \| 'heart failure'/exp \| 601824 \| \| #2 \| 'congestive cardiomyopathy'/exp \| 35723 \| \| #3 \| ((cardiac OR cardiocirculatory OR 'cardio circulatory' OR cardiogenic OR cardiomyopath* OR cardiopulmonary OR cardiovascular OR heart OR myocardial OR ventricle* OR ventricular) NEAR/3 (arrest OR collapse OR failure* OR insufficienc* OR decompensat* OR shock?)):ti,ab,kw \| 449782 \| \| #4 \| ((diastolic OR systolic OR ventricle? OR ventricular) NEAR/3 (dysfunction OR overload)):ti,ab,kw \| 73541 \| \| #5 \| 'heart edema*':ti,ab,kw OR 'heart oedema*':ti,ab,kw \| 41 \| \| #6 \| ((cardiorenal OR 'cardio renal') NEAR/3 syndrome):ti,ab,kw \| 2687 \| \| #7 \| #1 OR #2 OR #3 OR #4 OR #5 OR #6 \| 724706 \| \| #8 \| 'nutrition'/exp \| 2567327 \| \| #9 \| 'malnutrition'/exp \| 193043 \| \| #10 \| 'nutritional deficiency'/exp \| 301996 \| \| #11 \| calorie*:ti,ab,kw OR deficienc*:ti,ab,kw OR diet*:ti,ab,kw OR eat*:ti,ab,kw OR energy:ti,ab,kw OR fats:ti,ab,kw OR fatty:ti,ab,kw OR food:ti,ab,kw OR malnutri*:ti,ab,kw OR malnourish*:ti,ab,kw OR nutrient*:ti,ab,kw OR nutrition*:ti,ab,kw OR protein*:ti,ab,kw OR undernutri*:ti,ab,kw OR undernourish*:ti,ab,kw \| 6652494 \| \| #12 \| #8 OR #9 OR #10 \| 8389479 \| \| #13 \| 'frailty'/de \| 19947 \| \| #14 \| 'frail elderly'/de \| 11522 \| \| #15 \| fragility:ti,ab,kw OR frail*:ti,ab,kw OR debilit*:ti,ab,kw OR 'functionally impair*':ti,ab,kw \| 120528 \| \| #16 \| ((sof OR chs) NEAR/3 index*):ti,ab,kw \| 105 \| \| #17 \| #12 OR #13 OR #14 OR #15 \| 126136 \| \| #18 \| #7 AND #12 AND #17 \| 1162 \| \| #19 \| #18 NOT ('conference abstract'/it OR 'conference paper'/it OR 'conference review'/it) \| 799 \| | |

3. Web of Science Core Collection

| Interface: Clarivate Analytics  Editions = A&HCI , ESCI , SCI-EXPANDED , SSCI  Date of Search: 22 June 2022  Number of hits: 488 | Field labels   - TS/Topic = title, abstract, author keywords and Keywords Plus - NEAR/x = within x words, regardless of order - * = truncation of word for alternate endings   Note: the *Exact search*-function was used for all the searches |
| --- | --- |
| \| **#** \| **Query** \| **Hits** \| \| --- \| --- \| --- \| \| 1 \| TS=((cardiac or cardiocirculatory or cardio-circulatory or cardiogenic or cardiomyopath* or cardiopulmonary or cardiovascular or heart or myocardial or ventricle* or ventricular) NEAR/2 (arrest or collapse or failure* or insufficienc* or decompensat* or shock?)) \| 350,165 \| \| 2 \| TS=((diastolic or systolic or ventricle? or ventricular) NEAR/2 (dysfunction or overload)) \| 55,714 \| \| 3 \| TS=(heart edema? or heart oedema?) \| 72 \| \| 4 \| TS=((cardiorenal or cardio-renal) NEAR/2 syndrome) \| 1,998 \| \| 5 \| #4 OR #3 OR #2 OR #1 \| 378,566 \| \| 6 \| TS=(calorie* or deficienc* or diet* or eat* or energy or fats or fatty or food or malnutri* or malnourish* or nutrient* or nutrition* or protein* or undernutri* or undernourish*) \| 9,611,046 \| \| 7 \| TS=(fragility or frail* or debilit* or "functionally impair*") \| 99,409 \| \| 8 \| TS=((SOF or CHS) NEAR/2 index*) \| 62 \| \| 9 \| #8 OR #7 \| 99,424 \| \| 10 \| #9 AND #6 AND #5 \| 488 \| | |

6. Cinahl

| Interface: Ebsco  Date of Search: 22 June 2022  Number of hits: 153 | Field labels   - MH+ = exploded Cinahl Heading - MH = non exploded Cinahl Heading - MW = word in Cinahl Heading - TI = title - AB = abstract - Nx = within x words, regardless of order - * = truncation of word for alternate endings |
| --- | --- |
| \| # \| Query \| Results \| \| --- \| --- \| --- \| \| S1 \| (MH "Heart Failure+") \| 47,317 \| \| S2 \| TI ( ((cardiac OR cardiocirculatory OR "cardio-circulatory" OR cardiogenic OR cardiomyopath* OR cardiopulmonary OR cardiovascular OR heart OR myocardial OR ventricle* OR ventricular) N3 (arrest OR collapse OR failure* OR insufficienc* OR decompensat* OR shock?)) ) OR AB ( ((cardiac OR cardiocirculatory OR "cardio-circulatory" OR cardiogenic OR cardiomyopath* OR cardiopulmonary OR cardiovascular OR heart OR myocardial OR ventricle* OR ventricular) N3 (arrest OR collapse OR failure* OR insufficienc* OR decompensat* OR shock?)) ) \| 88,187 \| \| S3 \| TI ( ((diastolic OR systolic OR ventricle? OR ventricular) N3 (dysfunction OR overload)) ) OR AB ( ((diastolic OR systolic OR ventricle? OR ventricular) N3 (dysfunction OR overload)) ) \| 10,774 \| \| S4 \| TI ( "heart edema*" OR "heart oedema*" ) OR AB ( "heart edema*" OR "heart oedema*" ) \| 0 \| \| S5 \| TI ( ((cardiorenal or cardio-renal) N3 syndrome) ) OR AB ( ((cardiorenal or cardio-renal) N3 syndrome) ) \| 354 \| \| S6 \| S1 OR S2 OR S3 OR S4 OR S5 \| 106,766 \| \| S7 \| MW (deficienc* or diet* or eat* or energy or fats or fatty or nutrient* or nutrition* or protein*) \| 511,985 \| \| S8 \| (MH "Nutrition+") \| 179,712 \| \| S9 \| (MH "Nutrition disorders+") \| 147,993 \| \| S10 \| (MH "Food+") \| 195,776 \| \| S11 \| TI ( (calorie* or deficienc* or diet* or eat* or energy or fats or fatty or food or malnutri* or malnourish* or nutrient* or nutrition* or protein* or undernutri* or undernourish*) ) OR AB ( (calorie* or deficienc* or diet* or eat* or energy or fats or fatty or food or malnutri* or malnourish* or nutrient* or nutrition* or protein* or undernutri* or undernourish*) ) \| 622,296 \| \| S12 \| S7 OR S8 OR S9 OR S10 OR S11 \| 825,755 \| \| S13 \| (MH "Frail Elderly") \| 8,431 \| \| S14 \| (MH "Frailty syndrome") \| 3,507 \| \| S15 \| TI ( (fragility or frail* or debilit* or "functionally impair*") ) OR AB ( (fragility or frail* or debilit* or "functionally impair*") ) \| 28,920 \| \| S16 \| S13 OR S14 OR S15 \| 32,327 \| \| S17 \| S6 AND S12 AND S16 \| 153 \| | |
